# Comorbidity of poor sleep and primary headaches among nursing staff in north China

**DOI:** 10.1186/s10194-015-0571-z

**Published:** 2015-10-08

**Authors:** Yan Wang, Jingdan Xie, Fei Yang, Shiwen Wu, Hebo Wang, Xiaolan Zhang, Hua Liu, Xin Deng, Wei Xie, Shengyuan Yu

**Affiliations:** International Headache Center, Department of Neurology, Chinese PLA General Hospital, Fuxing Road 28, Haidian District, Beijing 100853 China; Department of Neurology, The General Hospital of Chinese Armed Police Forces, Beijing, 100039 China; Department of Neurology, Hebei General Hospital, Shijiazhuang, Hebei Province 050051 China

**Keywords:** Comorbidity, Poor sleep, Primary Headache, Migraine, Tension-type headache, Chronic daily headache, Nursing staff

## Abstract

**Background:**

Sleep disorders and primary headaches are both more prevalent among nursing staff than in the general population. However, there have been no reports about the comorbidity of poor sleep and primary headaches among nursing staff.

**Methods:**

Stratified random cluster sampling was used to select 1102 nurses from various departments in three hospitals in north China. Sleep quality was assessed with the Pittsburgh Sleep Quality Index (PSQI). The diagnosis of primary headaches including migraine, tension-type headache (TTH), and chronic daily headache (CDH) was based on the International Classification of Headache Disorders, 3rd edition (beta version) (ICHD-3-beta).

**Results:**

The response rate was 93 %. Among 1023 nurses, the prevalence of poor sleep was 56.7 %. Of these, 315 nurses (34.13 %) had poor sleep comorbid with primary headaches. The prevalence of poor sleep in the groups with CDH (82.1 %), migraine (78.9 %), and TTH (59.0 %) was significantly higher than that in the group without headaches (47.3 %) (all *P* < 0.05). Multivariate logistic regression revealed that rotating shifts and suffering headache were independent risk factors for poor sleep. Also, the 1-year prevalence of the three types of primary headache was significantly increased in the poor sleep group (migraine: 21.2 % *vs.* 7.2 %; TTH: 27.9 % *vs.* 24.9 %; CDH: 4.1 % *vs.* 1.1 %; *P* < 0.05). Compared with normal sleepers, nurses with poor sleep were 1.72 times more likely to have severe headache (OR: 1.72, 95 % CI: 1.14–2.57).

**Conclusion:**

Comorbidity of poor sleep and primary headaches among nursing staff is common. Therefore, sleep quality should be carefully evaluated in nurses with primary headaches.

## Background

Headache is a common neurological symptom experienced by almost everyone. A female excess in the prevalence of primary headache has been shown in previous epidemiological surveys [1]. In our previous study, the 1-year prevalence of primary headache disorders among nursing staff in mainland China was 45.3 %, of migraine 14.8 % (migraine with aura 3.4 %, migraine without aura 11.4 %), of tension-type headache (TTH) 26.2 %, and of chronic daily headache (CDH) 2.7 %, remarkably higher than in the general population. Also, that study revealed that working a greater number of night shifts was also associated with increased prevalence of primary headache [2]. Women are 1.3 to 2.0 times more likely to have poor sleep than are men [3, 4]. Several previous surveys concluded that working night shifts was associated with high levels of sleep problems and sleepiness [5–7]. Due to the special work schedule of the nursing profession, shift-related insomnia is very common [5]. According to a cross-sectional survey of nurses in Japan, the prevalence of insomnia (29.2 %) was three to four times higher in nurses than that in the general population [8]. Nursing staff, which primarily comprises women who are shift workers, were more likely to suffer headache and sleep problems [9, 10]. Meanwhile, sleep and headache disorders share elements of anatomy and physiology [11]. However, to date, no study has investigated the comorbidity rate of poor sleep and primary headache among nursing staff. Therefore, a questionnaire-based study on headache profiles and sleep conditions of nurses was conducted to evaluate the association of sleep quality and primary headache.

## Methods

### Ethics

The study protocol was approved by the Ethics Committee of the Chinese PLA General Hospital, Beijing. All participants provided written informed consent after receiving a detailed explanation of the purpose and design of the study.

### Procedure

This was an analysis of a data subset from a cross-sectional survey of “the prevalence of primary headache and its associated factors among nursing staff in north China,” which was conducted from December 2013 to June 2014 in three 3A hospitals in north China [2]. A stratified random cluster sampling method was used to complete this survey. Eight clinical departments were randomly selected from each hospital, and 1102 nurses were invited to participate in the survey. These methods are fully described elsewhere [2]. All participants filled out a structured questionnaire and were interviewed face to face to confirm diagnoses.

### Questionnaire

Each nurse completed a structured questionnaire covering demographic and socioeconomic data, headache characteristics over the previous year, and occupation-related factors [2]. The headache profile section of the questionnaire was validated for headache assessment and diagnosis in the general population [1, 12]. The headache diagnosis was made according to ICDH-3-beta [13].

### Pittsburgh sleep quality index

The Pittsburgh Sleep Quality Index (PSQI) assesses multiple dimensions of sleep over a 1-month time period [14]. The Chinese version (CPSQI) was used in the assessment of nurses’ sleep quality. This questionnaire is a sensitive, reliable, and valid measure of sleep quality that was used to determine whether the nurses were poor sleepers. Poor sleeper was defined by a CPSQI global score greater than 5 [15]. The CPSQI comprises seven components: (1) subjective sleep quality, (2) sleep latency, (3) sleep duration, (4) habitual sleep efficiency, (5) sleep disturbances, (6) use of sleeping medication, and (7) daytime drowsiness and dysfunction. The sum of the seven component scores yields one global score of subjective sleep quality (range 0–21), and higher scores represent poorer subjective sleep quality.

### Statistics

Data were processed using Epidata 3.1 and analysed using SPSS 17.0. Data are presented as frequency counts and descriptive statistics. Attack frequency and headache intensity are presented as medians, and categorical variables as numbers and percentages. The continuous variables were analysed using Student’s *t*-test and analysis of variance (ANOVA), and the categorical variables were analysed using Chi-square tests. Multivariate logistic regression was applied to identify adjusted odds ratios (AORs) with 95 % confidence intervals (CIs) for sleep disorders and different types of headache according to socio-demographic characteristics. Statistical significance was set at *P* < 0.05.

## Result

Among 1102 invited participants, 58 declined to participate in the survey, 21 submitted incomplete questionnaires, and 1023 completed the survey. The response rate was 93 %. All respondents were female, and the median age was 27 years.

**Fig. 1:**
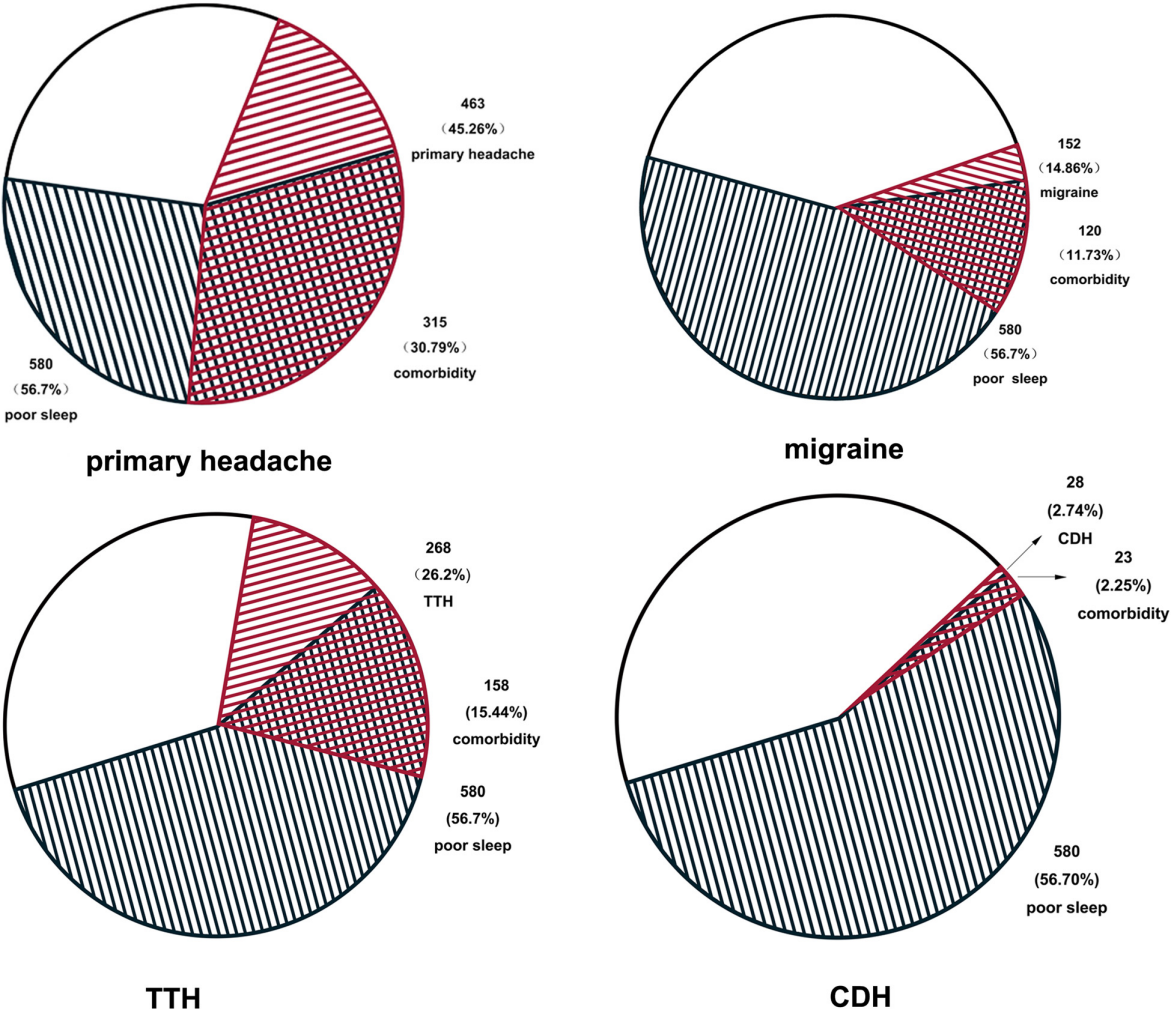
The comorbidity of primary headaches and poor sleep. The red line part represent the headache suffers, the black line part represent the poor sleep suffers and the grids part represent the comorbidity

Among 1023 nurses, 580 were assessed as having poor sleep quality. The 1-month prevalence of poor sleep was 56.7 % (95 % CI: 52.7–60.7 %). Of the 1023 participants, 315 suffered from both primary headache and poor sleep (comorbidity rate: 34.13 %), 120 suffered from both migraine and poor sleep (comorbidity rate: 11.73 %), 158 suffered from both TTH and poor sleep (comorbidity rate: 15.44 %), and 23 suffered from both CDH and poor sleep (comorbidity rate: 2.25 %)(see Fig. 1)

.

**Fig. 2:**
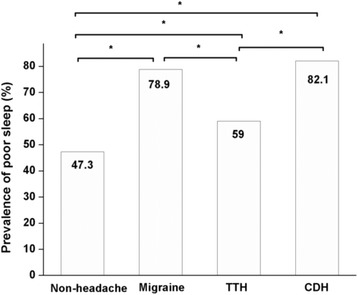
Comparison of the prevalence of poor sleep among nurses with different types of primary headaches and those without headaches.* *P* < 0.05

The prevalence of poor sleep in nurses with each of the three types of headaches is shown in Fig. 2. The prevalence in the CDH group was highest [82.1 % (95 % CI: 68.1–96.1 %)], followed by the migraine group [78.9 % (95 % CI: 72.4–85.4 %)], and was lowest in the TTH group [59 % (95 % CI: 53.1–64.9 %)].

Demographic data for normal sleepers and poor sleepers are shown in Table 1. Univariate analysis suggested that rotating-shift nurses were more likely to suffer poor sleep than were day-shift nurses (rotating-shift: 60 %, day-shift: 50.6 %, *P* < 0.01). Suffering headache significantly increased the probability of being a poor sleeper (68 % *vs.* 47.3 %, *P* < 0.01).

**Table 1:** Comparison of demographic characteristics among subjects between normal sleepers and poor sleepers

	Normal sleepers N (%)	Poor sleepers N (%)	*P*
Age			0.684
20–29	321 (43.1)	424 (56.9)	
30–39	86 (42.2)	117 (57.6)	
≥ 40	36 (48)	39 (52)	
Nationality			0. 259
Han	426 (43.7)	549 (56.3)	
Non-Han	17 (35.4)	31 (64.6)	
Marital status			0.465
Unmarried/Divorced	235 (42.3)	321 (57.7)	
Married	208 (44.5)	259 (55.5)	
Education			0.671
Junior colleague or lower	247 (40.3)	366 (59.7)	
University or above	196 (47.8)	214 (52.2)	
BMI			0.813
Normal (18.5- < 23)	283 (44.2)	357 (55.8)	
Lower than normal (<18.5)	65 (40.9)	94 (59.1)	
Overweight (23- < 25)	52 (44.1)	66 (55.9)	
Obese (≥25)	43 (40.6)	63 (59.4)	
Nursing specialty			0.570
Internal Medicine	197 (44.4)	247 (55.6)	
Surgical Department	158 (43.9)	202 (56.1)	
Others	88 (40.2)	131 (59.8)	
Title			0.743
Primary nurse	193 (43.5)	251 (56.5)	
Nurse practitioner	183 (42.3)	250 (57.7)	
Nurse-in-charge or above	67 (45.9)	79 (54.1)	
Work arrangement			0.003^**^
Day-shift	178 (49.4)	182 (50.6)	
Rotating-shift	265 (40)	398 (60)	
Headache			0.000^**^
No	295 (52.7)	265 (47.3)	
Yes	148 (59.2)	315 (68)	

*P < 0.05,**P < 0.01

The above factors were evaluated by multivariate logistic regression, which revealed that rotating shifts and suffering headache were independent risk factors for poor sleep. Rotating-shift work was associated with a 48.4 % increase in the incidence of poor sleep (AOR: 1.484, 95 % CI: 1.093–2.013, *P* < 0.05). Moreover, nurses with headache were more than two times more likely to suffer poor sleep than were nurses without headache (AOR: 2.518, 95 % CI: 1.931–3.283, *P* < 0.01).

**Fig. 3:**
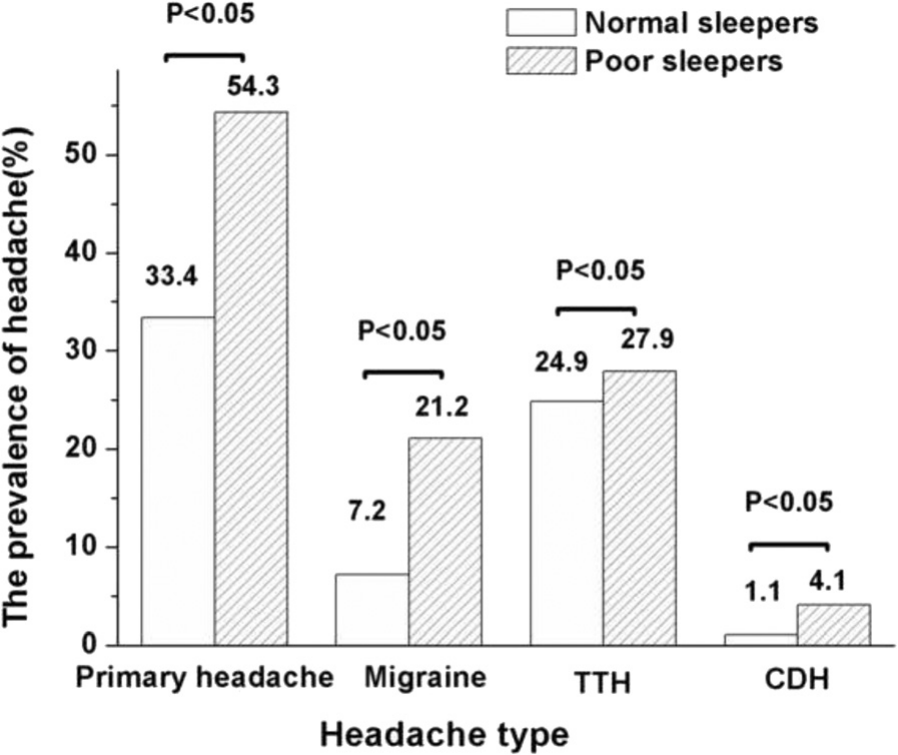
Comparison of the prevalence of different types of headache between normal sleepers and poor sleepers. White space represent the normal sleepers and black lines represent the poor sleepers

Figure 3 shows that the prevalence rates of all three types of primary headache were significantly increased in the group with poor sleep (migraine: 21.2 % *vs.* 7.2 %; TTH: 27.9 % *vs.* 24.9 %; CDH: 4.1 % *vs.* 1.1 %; *P* < 0.05). Demographic characteristics and sleep disturbance were analysed by multivariate logistic regression for the different types of primary headache, revealing that the poor sleepers were 2.734 times (95 % CI: 1.7–4.215) as likely to suffer migraine, 1.75 times (95 % CI: 1.22–2.505) as likely to suffer TTH, and 6.467 times (95 % CI: 2.3–17.97) as likely to suffer CDH.

The median attack frequency and headache intensity of the nurses who suffered from primary headaches were 2 days per month and 4, respectively. As shown in Table 2, poor sleep can influence headache frequency and intensity. Poor sleepers tended to have headache more frequently, but the difference was not significant. Compared with normal sleepers, nurses who suffered from poor sleep were 1.72 times more likely to have severe headache (OR: 1.72, 95 % CI: 1.14–2.57).

**Table 2:** The effect of poor sleep on headache frequency and headache intensity

Sleep	Headache frequency (episodes/month)			Headache intensity		
	≤2	>2	OR (95%CI)	VAS ≤ 4	VAS ≥ 4	OR (95%CI)
			1.48 (0.99–2.2)			1.72 (1.14–2.57)
Normal sleepers (N%)	91 (61.5 %)	57 (38.5 %)		98 (66.2 %)	50 (33.8 %)	
Poor sleepers (N%)	163 (51.9 %)	151 (48.1 %)		168 (53.3 %)	147 (46.7 %)	

## Discussion

This study, the first survey on the prevalence and factors associated with poor sleep among nursing staff in China, showed that the prevalence of poor sleep, as defined by a CPSQI score higher than 5, was 56.7 % [15], remarkably higher than the 8–30 % in the general population in China [3, 16]. By working rotating-shift schedules, individuals are exposed to work at the low point of their well-established circadian pattern, and their normal circadian systems are disrupted, which may ultimately result in poor sleep [7]. Many previous studies have shown that night work and an unstable shift schedule can impair mental health and lead to poor sleep [17–19]. Our finding showed that shift work was an independent risk factor for poor sleep, in agreement with previous epidemiologic studies. In Iran, of 925 health care workers, 43.1 % complained of sleep problems. [20] In addition, Takayuki reported that the insomnia prevalence among nursing staff was three to four times higher than that in the general population in Japan [8]. This is the first assessment of the comorbidity rate of different types of primary headache and poor sleep among nursing staff. No similar data have been reported previously.

The present study showed that the comorbidity of poor sleep with migraine and CDH was markedly greater than that with TTH. Additionally, compared with nurses without headaches, the comorbidity rate of poor sleep and all types of primary headache was higher. The results are similar to those of a previous study showing that headache sufferers had a high rate of poor sleep and that the prevalence varied for different headache types [21]. No large-scale epidemiological survey was available to describe sleep disorders and headache comorbidity rates in detail, but several small clinical studies have reached similar conclusions [22]. The prevalence of sleep disorders was 85.9 % among migraineurs, significantly higher than that in a group without headaches in the US state of Mississippi [23]. Also, Sancisi’s study suggested that CDH sufferers were 2.71 times more likely to have poor sleep than were episodic headache sufferers [24], but no distinction could be made for specific types of episodic headache. Our survey made up for this shortcoming and showed that the rate of comorbid poor sleep among CDH sufferers was higher than that among episodic TTH sufferers, but not higher than that in episodic migraineurs. In agreement with previous studies, our findings showed that poor sleep quality could increase the prevalence of primary headache. A survey including 310 community-dwelling Hong Kong Chinese women aged 40–60 years suggested that women with insomnia disorder had a 2.2-fold increased risk of reporting recurrent headache, a 3.2-fold increased risk of migraine, and a 2.3-fold increased risk of TTH, after adjusting for anxiety and depression [25]. In the present study, nurses with poor sleep were 2.229 fold as likely to suffer primary headache, 2.734 fold as likely to suffer migraine, 1.75 fold as likely to suffer TTH, and 6.467 fold as likely to suffer CDH, very similar to the results of the survey in Hong Kong. We also attempted to find out the effect of sleep quality on headache frequency and headache intensity. Previous research showed that headache of greater frequency was more strongly associated with insomnia than headache of lower frequency [25]. In our study, poor sleepers tended to experience headache more frequently, but the difference was not significant. The reason for this difference may be relatively small samples and different frequency grouping. Few studies have directly investigated the relationship between sleep disturbance and headache intensity, but previous studies have shown that poor sleep may reduce the pain threshold [26], leading to a more severe subjective feeling of headache. Our results supported this viewpoint. Our study had several strengths. First, it is the first study to assess the comorbidity of primary headaches and poor sleep in nursing staff. Second, it is the first study to confirm the bidirectional relationship between primary headache and poor sleep through epidemiological numerical values. Furthermore, the diagnosis of primary headaches met the latest ICDH-3-beta guidelines and was confirmed by a headache specialist.

Inevitably, there are several limitations in our study. Because of the limited conditions, we did not distinguish among specific types of sleep disorders. Also, we evaluated the prevalence and characteristics of primary headache over 1-year period, but assessed the sleep quality over a 1-month time period due to nurses’ limited memory for specific sleep conditions. In addition, previous studies suggested that shift-work-related sleep problems may be associated with several types of shifts (day shift, evening shift, and night shift) as well as with work schedules (different shift rotations) [5], but we did not distinguish among the specific shift types in our questionnaire

## Conclusion

The prevalence of poor sleep in nursing staff was remarkably higher than that in the general population. Rotating shifts are an independent risk factor for sleep disorders. Poor sleepers were more prone to suffer primary headache, especially severe headache than good sleepers. Moreover, nurses with primary headaches were more likely to be poor sleepers than were nurses without headache. Comorbidity of poor sleep and primary headaches among nursing staff is common. Therefore, sleep quality should be carefully evaluated in nurses with primary headache.

## Abbreviations

TTH: Tension type headache
CDH: Chronic daily headache
ICHD-3-beta: International Classification of Headache Disorders, 3rd edition (beta version)
CPSQI: Pittsburgh Sleep Quality Index (Chinese version)

## References

[1] Yu S, Liu R, Zhao G, Yang X, Qiao X, Feng J, Fang Y, Cao X, He M, Steiner T (2012). The prevalence and burden of primary headaches in China: a population-based door-to-door survey. Headache.

[2] Wang Y, Xie J, Yang F, Wu S, Wang H, Zhang X, Liu H, Deng X, Yu S (2015). The prevalence of primary headache disorders and their associated factors among nursing staff in North China. J Headache Pain.

[3] Li RH, Wing YK, Ho SC, Fong SY (2002). Gender differences in insomnia--a study in the Hong Kong Chinese population. J Psychosom Res.

[4] Reyner LA, Horne JA, Reyner A (1995). Gender- and age-related differences in sleep determined by home-recorded sleep logs and actimetry from 400 adults. Sleep.

[5] Flo E, Pallesen S, Akerstedt T, Mageroy N, Moen BE, Gronli J, Nordhus IH, Bjorvatn B (2013). Shift-related sleep problems vary according to work schedule. Occup Environ Med.

[6] Sallinen M, Kecklund G (2010). Shift work, sleep, and sleepiness - differences between shift schedules and systems. Scand J Work Environ Health.

[7] Akerstedt T (1998). Shift work and disturbed sleep/wakefulness. Sleep Med Rev.

[8] Kageyama T, Nishikido N, Kobayashi T, Oga J, Kawashima M (2001). Cross-sectional survey on risk factors for insomnia in Japanese female hospital nurses working rapidly rotating shift systems. J Hum Ergol (Tokyo).

[9] Lin KC, Huang CC, Wu CC (2007). Association between stress at work and primary headache among nursing staff in Taiwan. Headache.

[10] Nadaoka T, Kanda H, Oiji A, Morioka Y, Kashiwakura M, Totsuka S (1997). Headache and stress in a group of nurses and government administrators in Japan. Headache.

[11] Brennan KC, Charles A (2009). Sleep and headache. Semin Neurol.

[12] Yu SY, Cao XT, Zhao G, Yang XS, Qiao XY, Fang YN, Feng JC, Liu RZ, Steiner TJ (2011). The burden of headache in China: validation of diagnostic questionnaire for a population-based survey. J Headache Pain.

[13] The International Classification of Headache Disorders (2013). 3rd edition (beta version). Cephalalgia.

[14] Buysse DJ, Reynolds CF, Monk TH, Berman SR, Kupfer DJ (1989). The Pittsburgh Sleep Quality Index: a new instrument for psychiatric practice and research. Psychiatry Res.

[15] Tsai PS, Wang SY, Wang MY, Su CT, Yang TT, Huang CJ, Fang SC (2005). Psychometric evaluation of the Chinese version of the Pittsburgh Sleep Quality Index (CPSQI) in primary insomnia and control subjects. Qual Life Res.

[16] Chiu HF, Xiang YT, Dai J, Chan SS, Leung T, Yu X, Hou ZJ, Ungvari GS, Caine ED (2012). The prevalence of sleep problems and their socio-demographic and clinical correlates in young Chinese rural residents. Psychiatry Res.

[17] Oyane NM, Pallesen S, Moen BE, Akerstedt T, Bjorvatn B (2013). Associations between night work and anxiety, depression, insomnia, sleepiness and fatigue in a sample of Norwegian nurses. PLoS One.

[18] Akerstedt T (1988). Sleepiness as a consequence of shift work. Sleep.

[19] Muecke S (2005). Effects of rotating night shifts: literature review. J Adv Nurs.

[20] Ghalichi L, Pournik O, Ghaffari M, Vingard E (2013). Sleep quality among health care workers. Arch Iran Med.

[21] Tran DP, Spierings EL (2013). Headache and insomnia: their relation reviewed. Cranio.

[22] Rains JC, Penzien DB (2002). Chronic headache and sleep disturbance. Curr Pain Headache Rep.

[23] Walters AB, Hamer JD, Smitherman TA (2014). Sleep disturbance and affective comorbidity among episodic migraineurs. Headache.

[24] Sancisi E, Cevoli S, Vignatelli L, Nicodemo M, Pierangeli G, Zanigni S, Grimaldi D, Cortelli P, Montagna P (2010). Increased prevalence of sleep disorders in chronic headache: a case–control study. Headache.

[25] Yeung WF, Chung KF, Wong CY (2010). Relationship between insomnia and headache in community-based middle-aged Hong Kong Chinese women. J Headache Pain.

[26] Chiu YH, Silman AJ, Macfarlane GJ, Ray D, Gupta A, Dickens C, Morriss R, McBeth J (2005). Poor sleep and depression are independently associated with a reduced pain threshold. Results of a population based study. Pain.

